# Self-reported safety belt use among emergency department patients in Boston, Massachusetts

**DOI:** 10.1186/1471-2458-6-111

**Published:** 2006-04-27

**Authors:** William G Fernandez, Supriya D Mehta, Tara Coles, James A Feldman, Patricia Mitchell, Jonathan Olshaker

**Affiliations:** 1 Department of Emergency Medicine, Boston University School of Medicine, Boston, Massachusetts, USA

## Abstract

**Background:**

Safety belt use is 80% nationally, yet only 63% in Massachusetts. Safety belt use among potentially at-risk groups in Boston is unknown. We sought to assess the prevalence and correlates of belt non-use among emergency department (ED) patients in Boston.

**Methods:**

A cross-sectional survey with systematic sampling was conducted on non-urgent ED patients age ≥18. A closed-ended survey was administered by interview. Safety belt use was defined via two methods: a single-item and a multiple-item measure of safety belt use. Each was scored using a 5-point frequency scale. Responses were used to categorize safety belt use as 'always' or less than 'always'. Outcome for multivariate logistic regression analysis was safety belt use less than 'always'.

**Results:**

Of 478 patients approached, 381 (80%) participated. Participants were 48% female, 48% African-American, 40% White, median age 39. Among participants, 250 (66%) had been in a car crash; 234 (61%) had a valid driver's license, and 42 (11%) had been ticketed for belt non-use. Using two different survey measures, a single-item and a multiple-item measure, safety belt use 'always' was 51% and 36% respectively. According to separate regression models, factors associated with belt non-use included male gender, alcohol consumption >5 drinks in one episode, riding with others that drink and drive, ever receiving a citation for belt non-use, believing that safety belt use is 'uncomfortable', and that 'I just forget', while 'It's my usual habit' was protective.

**Conclusion:**

ED patients at an urban hospital in Boston have considerably lower self-reported safety belt use than state or national estimates. An ED-based intervention to increase safety belt use among this hard-to-reach population warrants consideration.

## Background

Motor vehicle occupant injuries represent the leading cause of injury mortality in the United States [1]. These preventable injuries are a major cause of cognitive and functional impairment, resulting in significant financial losses to both individuals and society [2]. The most effective means for motorists to reduce the risk of death and serious injury in a crash is the consistent use of safety belts [3]. Safety belt use has been shown to reduce motor vehicle occupant fatalities by 45%, and reduce the serious injury to the head, chest, and extremities by over 50% [4,5].

In the US, safety belt use among motorists is comparatively lower than that of other industrialized nations, such as Australia, Canada, Germany, and Great Britain [6,7]. One reason suggested as contributing to the lag in the US's safety belt use is the reliance on secondary rather than primary enforcement of mandatory safety belt legislation [6]. "Secondary enforcement" allows police to write a traffic citation for not wearing a safety belt only if a motorist is stopped for another violation (e.g., speeding). Primary enforcement laws have been shown to be more effective that secondary enforcement laws for increasing safety belt use [8]. In 2004, the overall rate of safety belt use in the US was 80% [9]. In the same year the prevalence of safety belt use among motorists in Massachusetts, a state with secondary safety belt enforcement, was 63%, the lowest in a national study of safety belt use [10]. Experts agree that the enforcement of primary safety belt legislation is the most effective measure to increase safety belt use. [3, 4, 5, 6, 7].

In addition to law enforcement measures, other multidisciplinary approaches targeting special populations (minorities, younger motorists, etc) are necessary to achieve and sustain high safety belt use levels [8]. Increasing safety belt use in the US is an important public health goal enumerated in the Healthy People 2010 objectives [11]. To emphasize this, the US Preventive Services Task Force has suggested that healthcare providers use their clinical encounters as an opportunity to discuss topics such as injury prevention with their patients [12]. Situated at the interface between the medical system and the public at large, emergency care personnel can play an important role in injury prevention [12]. In many parts of the US, the emergency department (ED) may serve as a "safety net" to treat the unmet health needs of a vulnerable segment of the population [14-16]. A disproportionate number of persons that use the ED as their primary source of care are minorities, or have lower levels of education and income [17-19]. The demographics of ED patients mirror the characteristics of individuals with lower rates of safety belt use [20-23]. As such, an ED visit could represent an important opportunity for providing harm-reduction counseling to patients in order to improve their safety belt use [14,15]. For example brief interventions have been successful in reducing the harmful consequences of alcohol, as well as helping motivate patients to abstain from heroin or cocaine [24-26]. Despite this, few studies have explored the correlates of the safety belt use and safety belt non-use among ED patients, a group which may exhibit more injury-prone behaviors than others, to determine if a harm-reduction intervention was warranted [27]. The goal of this project was to assess the prevalence of and factors associated with safety belt non-use among ED patients presenting for care at a public hospital in Boston.

## Methods

### Study design and setting

This was a cross-sectional study to determine the prevalence of and factors associated with safety belt non-use among ED patients at an urban, teaching hospital in Boston, Massachusetts. The facility has an annual volume of over 90,000 visits.

### Protocol

Research staff approached patients in the Adult ED through a process of systematic sampling (see below). Adult patients (age 18 and over) who were deemed "stable" by the ED attending on duty, with non-urgent medical complaints (i.e. patients not triaged to the acute area of the ED), who were able to speak either English or Spanish, and were able to provide verbal informed consent for this anonymous survey, were eligible for participation in the study. Patients were excluded if they were unable to provide verbal consent (including those experiencing altered mental status due to drug/alcohol intoxication, injury, or acute psychiatric illness); in severe pain or distress; if the patient was deemed by ED staff to be too ill to be interviewed (i.e., major trauma or medical illnesses); if the patient was a prisoner in custody, or if the patient had previously completed our survey. The Institutional Review Board at our institution approved this study.

### Sampling method

Our survey process consisted of systematically approaching adult ED patients via room-to-room assessment, between the hours of 8 am to 8 pm, Monday through Friday, from February 2004 to April 2004. The systematic sampling method consisted of research assistants walking clockwise from room-to-room in the ED, sampling every patient to identify those that met enrollment criteria. If a patient met enrollment criteria, they were asked to participate in a questionnaire on a range of health and safety issues important to emergency medicine practice. If a patient did not meet criteria, or refused to participate, research staff members went to the next sequential room.

### Study materials

Participants were asked questions read to them by research staff from a questionnaire in either English or Spanish. This was done to expedite survey completion, minimize the risk of missing data, and reduce the likelihood of language or literacy barriers. The survey contained questions regarding participants' demographics, frequency of riding in automobiles, frequency and correlates of safety belt use and safety belt non-use (e.g., listing items under the heading, 'things that make me wear my seat belt' and 'things that make me *not *wear my seat belt'), and frequency of alcohol consumption. The survey generally required 5 to 7 minutes to complete.

### Measures

The outcome of interest, safety belt use, was determined by two methods. Using a single, global measure of safety belt use, participants were asked to respond to the following single question based on a 5-point scale: "Think about the times you've ridden in a car in the past 30 days. Overall, how often did you wear a seatbelt? a) Always, b) More than half the time, c) About half the time, d) Less than half the time, or e) Never." According to the Single Measure, the overall safety belt use outcome for analysis was determined by the frequency of participants that answered 'always' on this question. Conversely, any answer other than 'always' on this scale was determined to be non-use. The Single Measure of Safety Belt Use is a validated single-item measure of safety belt use which has been validated, and is currently the standard instrument used for population-based surveys on safety belt use and other health-related risk factors in the US [28-31]. These population-based surveys are used to monitor the behaviors that may influence disease or injury, as well as to help set the national health agenda. Despite its wide use in survey-based research, the Single Measure of Safety Belt Use has its limitations. Critics point out that the use of the single measure can overestimate observed safety belt use by 2–27% as compared to direct observation [32-34]. For example, in a study of self-reported safety belt use compared to directly observed safety belt use, Nelson found that the validity of self-reported safety belt use was best among respondents residing in States with the highest observed prevalence of safety belt use, and conversely, the lowest validity was in States with a low prevalence of safety belt use [32]. He suggests that the risk of over-reporting bias due to social desirability would be greatest in areas of lowest observed safety belt use. Also, in the 2003 Motor Vehicle Occupant Safety Survey sponsored by the National Highway Transit Safety Administration, self reported safety belt use using a single measure was 84% [35]. However, in a follow-up question, 7% of persons stating they wore their safety belt 'always' stated they had driven unbelted within the past week. Using these two questions in previous years' Motor Vehicle Occupant Safety Survey, the follow-up question reduced the rate of self-report safety belt use by an average of 8–10%.

**Figure 1:**
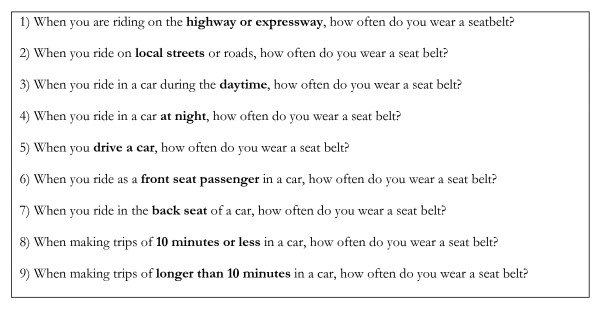
The Multiple Measure of Safety Belt Use. Response categories were: 'Always', 'More than half the time', 'Half the time', 'Less than half the time', 'Never'.

To minimize the effects of over-reporting in our study, safety belt use was also assessed by a series of nine separate questions (the Multiple Measure of Safety Belt Use) on scenario-specific aspects of transportation (highway, local, daytime, nighttime, driver, front-seat passenger, backseat, short rides, and long rides) (Figure 1). We constructed the Multiple Measure of Safety Belt Use – an instrument consisting of 9 distinct scenario-specific survey questions – via a focus group of public health researchers and emergency physicians. We piloted the questions among a sample of ED patients for content and syntax prior to commencing the study. Participants were prompted to answer these questions using the same 5-point frequency scale used to assess the Single Measure of Safety Belt Use. A description of the Multiple Measure of Safety Belt Use, and a comparison between the Single Measure and the Multiple Measure has been presented elsewhere [35]. Briefly, we classified safety belt use by the Multiple Measure of Safety Belt Use as a response of 'always' to all nine scenarios. Participants that responded with an answer of less than 'always' to any one of the above 9 questions were categorized as non-use according to the Multiple Measure.

### Statistical analysis

The explanatory variables that were examined in association with the outcome were: demographics (age, gender, race, education, possession of a valid driver's license); safety questions (history of a car crash, being cited for not wearing a safety belt, drinking alcohol prior to driving a car, riding in a car with someone who drank alcohol before driving); frequency of use of public and private transportation; and attitudes about seatbelt use ('seatbelts are uncomfortable', etc.).

To assess factors associated with seatbelt non-use, chi-square test was performed for categorical variables. Variables that were statistically significant at the p < 0.05 level from exploratory analysis were tested via univariate logistic regression. Statistically significant variables from univariate logistic regression (p < 0.05) were used to construct a multivariate logistic regression model. Backwards likelihood ratio testing was employed for model selection. Data was analyzed using STATA/SE 8.0 for Windows (Stata Corporation, College Station, TX).

## Results

### Characteristics of study subjects

Of 478 individuals approached, 39 (8%) patients refused, 58 (12%) were excluded, and 381 (80%) patients completed the survey. Patients were excluded for the following reasons: pain/too sick (18), language barrier (22), in custody (6), altered mental status (9), previously enrolled (3). There were 183 (48%) African Americans, 10 (3%) Asians, 2 (1%) Native American, 151 (40%) Whites, and 34 (9%) Other patients enrolled in the study. In terms of ethnicity, there were 77 (20% of the total sample) Hispanic or Latino, 19 (5%) Haitian, and 20 (5%) Cape Verdean. For the purpose of this paper, the term 'Hispanic' will be used to refer to those persons self-described as 'Hispanic or Latino'. Of the 77 Hispanics in the study, 32 (8% of total sample) completed their surveys in Spanish. Although not generally included, categories for 'Haitian' and 'Cape Verdean' ethnicity were added because they represent some of the main ethnic groups represented at our institution. The mean age of study participants was 39 years. Patients were 52% male and 48% female (two surveys had missing responses to gender), and 78% of respondents had at least a high school education. There were no statistical differences between participants and those that elected not to participate in terms of age or sex, however, enrollment was only 36% among Asians, compared to 100% for Whites, 87% for African-Americans, and 87% of other races (p < 0.001). Also, Hispanics were less likely to enroll than patients of other ethnicities (79% Hispanic vs. 100% Cape Verdean, 95% Haitian, p = 0.007).

### Main results

Table 1 shows the association between demographic characteristics and responses to the Single Measure and the Multiple Measure of Safety Belt Use. Women and ethnicity were statistically associated with a response of 'always' wearing a safety belt. Table 2 shows the results of selected questions on safety belt use. Some items associated with belt use were: a history of being in a car crash, receiving a citation for not wearing a safety belt, as well as the quantity and frequency of alcohol use.

Table 3 shows the variance between the Single Measure and the Multiple Measure of Safety Belt Use. The greatest variance between an answer of 'always' on the Single Measure and the specific scenarios of the Multiple Measure of Safety Belt Use was being a back seat passenger (147 of 187, 79% overall agreement); the greatest agreement between the Single Measure and specific scenarios was in driving (137 of 142 respondents able to operate a vehicle, 97% overall agreement). According to the Single Measure of Safety Belt Use, 192 of 382 respondents (51%) stated the 'always' wear safety belts. However, using the 9 components of the Multiple Measure of Safety Belt Use, only 137 of 382 (36%) indicated they 'always' wear safety belts. The converse situation was not observed, where respondents reported less than 'always' to the Single Measure, but 'always' to all nine items of the Multiple Measure.

Reasons were elicited for participants' safety belt use and non-use, and were compared to responses on the Single Measure and Multiple Measure of Safety Belt Use. The statements that "seatbelts are too uncomfortable" and "I just forgot" were among those most strongly associated with safety belt non-use (i.e. "things that make me *not *wear my seatbelt") on both the Single Measure and the Multiple Measure of Safety Belt Use (p < 0.001). The statements, "I like taking risks" and "I've got too many other things to think about" were strongly associated with non-use according to the Single Measure (p < 0.001). The statements "I've got too many other things to worry about" and "friends make fun of me when I wear a seatbelt" were also associated with non-use according to the Multiple Measure (p = 0.009 and p = 0.016, respectively). The statement that "wearing my seatbelt is my usual habit" was among those that were most strongly associated with safety belt use (i.e., "things that make me wear my seatbelt") according to both the Single Measure and the Multiple Measure of Safety Belt Use (p < 0.001). The statement "I'm afraid of being killed in a car crash" was also associated with safety belt use according to both the Single Measure and Multiple Measure of Safety Belt Use (p = 0.005 and p = 0.009, respectively) The statements "I wear my seat belt because it's the law", "my parents made we wear a seatbelt" and "everyone I know wears a seatbelt" were all strongly associated with safety belt use according to the Single Measure (p < 0.001, p = 0.002, p = 0.003, respectively).

The results of a multivariate logistic regression model of factors associated with less than 'always' wearing a safety belt according to the Single Measure of Safety Belt Use are shown in Tables 4. Male gender, maximum alcohol consumption of greater than 5 drinks in a single episode, riding with others that drink and drive, ever receiving a traffic citation for not wearing a safety belt, the belief that safety belt use is "uncomfortable", and that "I just forget to use my seatbelt" were risks for safety belt non-use, while "it's my usual habit" was protective. Table 5 shows the results of a multivariate logistic regression model predicting less than 'always' wearing a safety belt according to the Multiple Measure of Safety Belt Use. Risks included: male gender, use of public transportation greater than 10 times per month, riding with others that drink and drive, being given a traffic citation for not wearing a safety belt, the belief "wearing a seatbelt is uncomfortable", that "I've got too many other things to think about", and "I just forget to use my seatbelt". As in the previous model, a response that safety belt use is a usual habit was protective against non-use.

## Discussion

In this study, we sought to assess the prevalence, correlates, and barriers to safety belt use among ED patients at an urban public hospital in Boston, Massachusetts. Self-reported safety belt use among ED patients in our sample was even lower than state-level norms obtained by direct observation [10]. According to a single-item query, the prevalence of consistent safety belt use was 51%; though using a Multiple Measure, safety belt use was only 36%. In separate multivariate regression models, factors associated with safety belt non-use were male gender, increased alcohol consumption, riding with others that drink and drive, being cited for not wearing a safety belt, the belief that safety belt use is 'uncomfortable', that 'I just forgot', while reporting that 'safety belt use is my usual habit' was protective of non-use. In a regression model using the Multiple Measure, an additional factor associated with safety belt non-use was the frequent use of public transportation.

According to both measures of safety belt use, females were more likely than males to report that they always wear a seat belt. This corresponds to previous research demonstrating that males report lower seat belt use [20,23,36]. Respondents who reported drinking and driving in the past 30 days were less likely to wear seat belts. Previous investigations have noted that excessive alcohol use is correlated with other high-risk behaviors, including seat belt non-use [37,38]. Respondents that had ever received a traffic citation for not wearing a safety belt, or who stated that seatbelts are uncomfortable or that they forget to put on the seat belt were more likely to report seat belt non-use. Conversely, those that stated that wearing a seat belt is a usual habit or that stated that they were afraid of being in a car crash were more likely to report seat belt use. In a national survey by the National Highway Traffic Safety Administration, 96% of motorists cited 'avoiding serious injury', 84% cited 'wearing a safety belt is my usual habit', and 71% cited 'I don't want to get a ticket' as primary reasons for safety belt use, and some of the most cited reasons for safety belt non-use among motorists were 'I forgot' (53%), and 'seat belts are uncomfortable' (37%) [31].

Using the Multiple Measure, an additional predictor of seat belt non-use was frequent use of public transportation. We hypothesize that among ED patients in Boston, Massachusetts, those with higher frequency of public transportation may have fewer occasions to ride in passenger vehicles, which may lead to an increase likelihood of over-reporting safety belt use behavior on a standard survey questionnaire. In a bivariate analysis, persons of Hispanic and Haitian ethnicity had higher safety belt use than Whites, according to the Single Measure. This is surprising, as non-white race and Hispanic ethnicity has been shown to be associated with non-use [21,39,40]. It is important to note that in each of these studies, a single question was used to determine self-reported safety belt use. As demonstrated in our study, the Single Measure may have led to an over-estimate of self-reported safety belt use. Future research using our proposed Multiple Measure could examine cultural differences in terms of response bias. Our study did not find an association between safety belt use and race/ethnicity, age, or educational levels by either of the two survey measures. Since our study was carried out among patients presenting for care at an urban public hospital, we did not assess income level, as we made the assumption that our patient sample had lower income. Lower income has been shown to be associated with lower levels of safety belt use in other studies [20,23,40]. A number of risk taking behaviors have also been shown to be associated with safety belt non-use, such as excessive alcohol use, unsafe driving, and marijuana use [20,37,38,41,42]. Overall, a certain profile emerges (males, those who consume greater than 5 drinks on a single occasion, ride in vehicles with others that drink and drive, or have been cited previously for not wearing a safety belt, and those who "just forget" to wear safety belts, or find them "uncomfortable) that represent a high-risk group that may benefit from an ED based targeted intervention.

We used two methods to estimate overall safety belt use a commonly used measure, the Single Measure, and a multi-item Multiple Measure of Safety Belt Use [14]. We hypothesized that a single global measure may be more vulnerable to over-reporting. A situation where participants indicated their belt use was other than 'always' on the Single Measure, but answered 'always' to all 9 items of the Multiple Measure of Safety Belt Use (ostensibly, the *false negative *result) did not occur. According to state-level observational data, the rate of safety belt use is highest for drivers, and lowest for rear-seat passengers. In the 1998 Motor Vehicle Occupant Safety Study, 79% of respondents reported wearing their safety belts while driving, while only 43% of respondents reported wearing their safety belt while riding as a passenger in the back seat [31].

We used a Multiple Measure of Safety Belt Use that, by design, was a more stringent measure of self-reported safety belt use. This is because previous research suggested that over-reporting increases with a Single Measure as levels of observed safety belt use decrease [32]. We feel that for large, population-based surveys, the Single Measure of Safety Belt Use will continue to be the standard public health research instrument. However, if the Multiple Measure of Safety Belt Use can be validated in future studies, it may render a more accurate estimate of actual safety belt use than would the Single Measure in smaller community-level settings – such as neighborhoods with historically low safety belt use – when an observational study is not financially or logistically feasible. Additionally, it helps to better define scenarios in which safety belts are not worn. Overall, our results suggest that a sample of ED patients in Boston, Massachusetts, have low self-reported rate of safety belt use. Based on these results, additional work in order to create an ED-based intervention to increase safety belt use in this hard-to-reach population is warranted.

## Limitations

This study had some limitations. First, the generalizability of our findings to the general population may be limited, as this was a clinical population. Clinical populations can differ from population-based samples in terms of health and other factors [43-45], and may be different in terms of safety belt use. In particular, it has been reported that ED patients have higher injury-prone behaviors than other clinical populations [27]. This, in turn, would provide an opportunity for conducting a targeted intervention to increase safety belt use among an injury-prone population with low self-reported safety belt use. Despite the limitations in this study, it is apparent that a harm-reduction intervention, using the methods of other interventions successful in reducing alcohol and substance misuse, would be justified in the ED setting. Additionally, the rate of participation by race and ethnicity was unequal, and as such, the study may have suffered from volunteer bias. There may have been a response bias resulting from research assistants asking the survey questions in a face-to-face interview instead of having participants complete a self-reported survey. The lack of Hispanic or African-American research assistants may have altered the rate of participation. Survey-based methods of assessing safety belt use often suffer from some degree of overestimation of actual safety belt use, compared with observational methods. However, as noted above, safety belt use among respondents was far below both the state- and national-level measurements from observational studies [9,10]. Based on our study design, we feel that over-reporting of safety belt use behavior by respondents was minimized.

## Conclusion

In summary, the current study demonstrates that ED patients at an urban public hospital in Boston, Massachusetts, have self-reported safety belt use which is quite low, and considerably lower than state or national averages. The findings from this project support further work to develop a targeted intervention to increase safety belt use among this hard-to-reach population.

## Competing interests

The author(s) declare that they have no competing interests.

## Authors' contributions

WGF, SDM and JF conceived and designed the study. JF and PM provided guidance with IRB approval. PM supervised the data collection, as well as managed the data. SDM analyzed the data. TC and JO participated in writing the initial manuscript. All the authors contributed to piloting the survey instrument, interpreting the data, and editing the manuscript. All authors have read and approved of the final manuscript. WGF takes responsibility for the paper as a whole.

## Pre-publication history

The pre-publication history for this paper can be accessed here:



## Figures and Tables

**Table 1:** Demographic data, response to global and multiple measures of safety belt use among urban emergency department patients

**Variable**	**Total Sample N (%)**	**SBU 'Always', by Single Measure of Safety Belt Use N (%)**	**Chi Square**	**P value**	**SBU 'Always', by Multple Measure of Safety Belt Use N (%)**	**Chi Square**	**P value**
*Gender*							
Male	197 (52)	80 (42)	15.7	<0.001	35 (36)	14.4	<0.001
Female	182 (48)	111 (58)			63 (64)		
*Age*							
18–24	49 (13)	26 (14)	7.68	0.958	17 (17)	2.63	0.622
25–34	98 (26)	46 (24)			23 (23)		
35–44	108 (28)	51 (27)			28 (29)		
45–54	81 (21)	42 (23)			19 (19)		
55 or older	46 (12)	27 (14)			11 (11)		
*Race*							
African American	183 (48)	91(47)	7.40	0.116	55 (53)	3.05	0.549
Asian	10 (3)	6 (3)			1 (1)		
Native American	2 (1)	2 (1)			1 (1)		
White	151 (40)	70 (36)			37 (38)		
Other	34 (9)	23 (12)			7 (7)		
**Ethnicity**							
Hispanic or Latino	77 (20)	50 (26)	14.9	0.002	23 (23)	2.02	0.568
Cape Verdean	19 (5)	9 (5)			3 (3)		
Haitian	20 (5)	15 (8)			4 (4)		
None of These	264 (69)	118 (61)			68 (69)		
**Education**							
< High School	27 (7)	16 (8)	4.77	0.312	7 (7)	7.32	0.12
Some High School	55 (15)	22 (12)			8 (8)		
High School/GED	136 (36)	65 (34)			32 (33)		
Some College	91 (24)	50 (26)			27 (27)		
College Graduate	70 (18)	38 (20)			24 (24)		

**SBU **= Safety belt use

**Single Measure of Safety Belt Use **= Respondents were asked the following question: "Think about the times you've ridden in a car in the *past 30 days*. Overall, how often did you wear a seatbelt?" The overall outcome of analysis, safety belt use, was determined by the frequency of participants that answered 'always' on this question.

**Multiple Measure of Safety Belt Use **= Respondents were asked a series of situation-specific transportation questions (highway travel, local road travel, daytime travel, travel at night, driving a car, front seat passenger, back seat passenger, short trips of 10 minutes or less, and trips longer than 10 minutes). We classified safety belt use via the Multiple Measure as a response of 'always' to all nine scenarios.

**Table 2:** Responses to selected questions on safety belt use

Variable	Total Sample N (%)	SBU 'Always', by Single Measure N (%)	Chi Square	P value	SBU 'Always', by Multiple Measure N (%)	Chi Square	P value
Ever been in crash?							
Yes	250 (66)	109 (57)	14.03	<0.001	60 (61)	1.04	0.308
No	130 (34)	83 (43)			38 (39)		
							
Driving after drinking (in past 30 days)?							
Yes	24 (6)	5 (3)	9.57	0.008	3 (3)	22.17	<0.001
No	298 (78)	154 (80)			93 (95)		
I don't drive	58 (15)	33 (17)					
							
Ride with someone who drove after drinking (in past 30 days)?							
Yes	57 (15)	14 (7)			9 (9)	3.419	0.064
No	323 (85)	178 (93)			89 (91)		
							
Ever given ticket for not wearing a seatbelt?							
Yes	42 (11)	9 (5)	15.9	<0.001	4 (4)	6.84	0.033
No	337 (89)	182 (95)			94 (96)		
							
How often did you use public transportation in past 30 days?							
10+ times	184 (48)	89 (46)	1.02	0.795	31 (32)	15.7	0.001
5–9 times	23 (6)	11 (6)			7 (7)		
1–4 times	49 (13)	25 (13)			16 (16)		
Not in past 30 days	124 (33)	67 (35)			44 (45)		
							
On average, how many drinks do you have on a typical day?							
0–1 drinks	228 (60)	131 (68)	14.053	<0.001	66 (67)	6.847	0.033
2–5 drinks	110 (29)	49 (26)			28 (29)		
6 + drinks	42 (11)	12 (6)			4 (4)		
							
Maximum # of drinks on a given day (in past 30 days)?							
0–1 drinks	223 (59)	130 (68)	17.52	<0.001	64 (65)	9.94	0.007
2–5 drinks	95 (25)	44 (23)			28 (29)		
6 or more drinks	62 (16)	18 (9)			6 (6)		

**SBU **= Safety Belt Use

**Table 3:** Agreement between responses of 'always' on a Single Measure and specific scenarios of the Multiple Measure of Safety Belt Use

Situation where response was 'Always'	Number responding 'Always' on Single Measure	Number responding 'Always' using Multiple Measure	Percent Agreement
Highway travel	191	184	96%
Local road travel	192	178	93%
Daytime travel	192	179	93%
Nighttime travel	192	181	95%
Driving*	143	139	97%
Front-seat passenger	191	175	92%
Back-seat passenger	187	147	79%
Short trips (<10 min)	191	165	86%
Long trips (10+ min)	191	182	95%

* Respondents indicating that they do not drive were excluded

**Table 4:** Multivariate logistic regression model: Factors associated with safety belt use less than always using a Single Measure of Safety Belt Use

Variable	Odds Ratio	P value	95% Confidence Interval	
Male Gender	2.18	0.009	1.22	3.91
I just forget to wear my seatbelt*	2.55	0.002	1.42	4.55
Rides with others who drink and drive	3.21	0.008	1.67	7.57
Ever received a ticket for not wearing a seatbelt	4.06	0.003	1.63	10.1
Maximum alcohol consumption Single Episode^^ ^>5 drinks	2.83	0.014	1.23	6.50
Wearing a seatbelt is a usual habit*	0.03	<0.001	0.01	0.08
Wearing a seatbelt is uncomfortable*	4.12	<0.001	2.26	7.54

* Reference category is "Not at All"; Indicator is "A little" or "A lot"

^^ ^Reference is "0–1 drink"

**Table 5:** Multivariate logistic regression model: Factors associated with safety belt use less than always using a Multiple Measure of Safety Belt Use

Variable	Odds Ratio	P value	95% Confidence Interval	
Male Gender	2.18	0.008	1.23	3.89
I just forget to wear my seatbelt*	3.07	<0.001	1.69	5.57
Rides with others who drink and drive	3.18	0.016	1.24	8.16
Ever received a ticket for not wearing a seatbelt	3.42	0.028	1.14	10.3
Uses public transportation ≥ 10 times per month ^$^	2.64	0.001	1.46	4.80
Wearing a seat belt is uncomfortable*	5.45	<0.001	2.74	10.8
Wearing a seatbelt is a usual habit*	0.07	<0.001	0.02	0.23
I have too many other things to think about*	3.56	0.037	1.08	11.8

* Reference category is "Not at All"; Indicator is "A little" or "A lot"

^$ ^Reference category is "< 10 times per month"
